# 

**Published:** 1881-08

**Authors:** 


### CONTENTS.

### COMMUNICATIONS.

"Oral Electricity and the New Departure" ........................ 315
Prosthetic Dentistry ........................................................ 330
Annual Meeting of the Illinois State Dental Society ........................ 337

### SELECTIONS.

The Use of Tobacco ........................................................ 343
Natural History Notes ...................................................... 344
Neuralgia as a "Warning" ................................................... 345
Suggestions Concerning Long Life ............................................ 346
Cincinnati Academy of Medicine .............................................. 346

CORRESPONDENCE .............................................................. 350

### EDITORIAL.

A Singular Case ............................................................ 351
Alumni Matters .............................................................. 352
Commencement ................................................................ 353
The American Dental Association ............................................. 353
Off for Europe .............................................................. 354
American Dental Microscopists ............................................... 354
Obituary .................................................................... 354

